# Incidence of Carbapenem-Resistant Gram-Negative Bacterial Infections in Critically Ill Patients with COVID-19 as Compared to Non-COVID-19 Patients: A Prospective Case-Control Study

**DOI:** 10.1155/2024/7102082

**Published:** 2024-06-22

**Authors:** Diamanto Aretha, Sotiria Rizopoulou, Leonidia Leonidou, Sotiria Kefala, Vasilios Karamouzos, Maria Lagadinou, Anastasia Spiliopoulou, Markos Marangos, Fotini Fligou, Fevronia Kolonitsiou, Fotini Paliogianni, Stelios F. Assimakopoulos

**Affiliations:** ^1^ Department of Anesthesiology and Intensive Care Medicine University of Patras Medical School, Patras, Greece; ^2^ Department of Internal Medicine University of Patras Medical School, Patras, Greece; ^3^ Department of Microbiology University of Patras Medical School, Patras, Greece

## Abstract

**Introduction:**

Critically ill COVID-19 patients hospitalized in intensive care units (ICU) are immunosuppressed due to SARSCoV-2-related immunological effects and are administered immunomodulatory drugs. This study aimed to determine whether these patients carry an increased risk of multi-drug resistant (MDR) and especially carbapenem-resistant Gram-negative (CRGN) bacterial infections compared to other critically ill patients without COVID-19.

**Materials and Methods:**

A prospective case-control study was conducted between January 2022 and August 2023. The ICU patients were divided into two groups (COVID-19 and non-COVID-19). Differences in the incidence of CRGN infections from *Klebsiella pneumonia*e, *Acinetobacter* spp., and *Pseudomonas aeruginosa* were investigated. In addition, an indicator of the infection rate of the patients during their ICU stay was calculated. Factors independently related to mortality risk were studied.

**Results:**

Forty-two COVID-19 and 36 non-COVID-19 patients were analyzed. There was no statistically significant difference in the incidence of CRGN between COVID-19 and non-COVID-19 patients. The infection rate was similar in the two groups. Regarding the aetiological agents of CRGN infections, *Pseudomonas aeruginosa* was significantly more common in non-COVID-19 patients (*p*=0.007). COVID-19 patients had longer hospitalisation before ICU admission (*p*=0.003) and shorter ICU length of stay (LOS) (*p*=0.005). ICU COVID-19 patients had significantly higher mortality (*p* < 0.001) and sequential organ failure assessment (SOFA) score (*p* < 0.001) compared to non-COVID-19 patients. Μortality secondary to CRGN infections was also higher in COVID-19 patients compared to non-COVID-19 patients (*p*=0.033). Male gender, age, ICU LOS, and hospital LOS before ICU admission were independent risk factors for developing CRGN infections. Independent risk factors for patients' mortality were COVID-19 infection, obesity, SOFA score, total number of comorbidities, WBC count, and CRP, but not infection from CRGN pathogens.

**Conclusions:**

The incidence of CRGN infections in critically ill COVID-19 patients is not different from that of non-COVID-19 ICU patients. The higher mortality of COVID-19 patients in the ICU is associated with higher disease severity scores, a higher incidence of obesity, and multiple underlying comorbidities, but not with CRGN infections.

## 1. Introduction

A minority of patients with COVID-19, usually older patients, with underlying comorbidities or immunosuppression, might develop a severe clinical course associated with acute respiratory distress syndrome (ARDS) and multiple organ failure. These critically ill patients are supported with mechanical ventilation in intensive care units (ICU). Pathophysiologically, the severe or critical illness from COVID-19 is characterized by a “cytokine storm” with excessive release of proinflammatory mediators [1]. Therefore, therapeutically dexamethasone and biological factors, such as tocilizumab (an IL-6 inhibitor), baricitinib (a Janus kinase inhibitor), or anakinra (an IL-1 inhibitor) are used [2, 3]. However, these agents have been associated with an increased risk of severe bacterial infections [4, 5].

Critically ill patients are at high risk of developing hospital-acquired infections, mainly ventilator-associated pneumonia (VAP) and bloodstream infections [6, 7]. Prior colonization with resistant pathogens, surgery during current admission, prior dialysis with end stage renal disease, use of carbapenems, and stay in the ICU for more than 5 days have been identified as independent risk factors for multi-drug resistant (MDR), especially carbapenem-resistant Gram-negative (CRGN), bacterial infections [8]. This risk of nosocomial ICU-acquired infections is increased in COVID-19 patients due to frequent, significant, and prolonged lymphopenia and the use of glucocorticosteroids or biological factors that promote immunosuppression [9]. Amongst these infections developed in critically ill COVID-19 patients, a proportion ranging between 32% and 50% is caused by MDR pathogens [10–13]. *Enterobacteriaceae*, *Pseudomonas aeruginosa*, and *Acinetobacter* spp. are common Gram-negative resistant pathogens that account for bacterial superinfections in COVID-19 patients [9, 11, 14, 15]. However, most previous studies estimating the risk of MDR infections in COVID-19 patients did not include a comparative non-COVID-19 group of patients to counterbalance the contribution of the ICU epidemiology, which is especially necessary in ICUs with a high prevalence of MDR isolates [16].

This study aimed to determine whether critically ill ICU patients suffering from COVID-19 infection carry an increased risk of CRGN bacterial infections compared to critically ill patients without COVID-19. This risk was also estimated relative to the time spent in the ICU for each patient. The study's secondary objectives were to identify differences between the two groups of patients in baseline characteristics, disease severity and outcome, and identify factors related to CRGN infection and mortality risk.

## 2. Materials and Methods

### 2.1. Patients

This prospective case-control study was conducted at the ICU of Patras General University Hospital, a tertiary, academic, 750-bed hospital, between January 2022 and August 2023. The study protocol was approved by the Hospital Research Ethics Committee (PN: 10408), and the need for informed consent was waived. During the pandemic, informed consent was waived for all observational studies, according to our hospital's Research Ethics Committee, in order to limit the spread of the disease. However, prior to the patient's enrollment in the study, there was a phone call to the legal representative, and permission to collect data was requested.

Patients' primary clinical and demographic data were retrospectively collected from the electronic clinical records on the day of ICU admission. From that day onwards, the data of interest were collected prospectively, and survival was assessed at ICU discharge. The patients were divided into two groups (COVID-19 and non-COVID-19).

The criteria for inclusion in the study were patients aged >18 years who were hospitalized in the ICU during the study and who developed after 48 h of ICU admission a nosocomial infection, which was microbiologically investigated by cultures of relevant samples and at least one set of blood cultures. All the patients that were included in the COVID-19 group suffered from pneumonia and mild or severe ARDS. The exclusion criteria were patients missing clinical or microbiological data and patients from whom blood culture samples were not taken or lost. All patients with positive cultures for CRGN pathogens were seen by an experienced infectious diseases specialist, and cases considered colonization were excluded. The following characteristics of patients were recorded: age, gender, type of CRGN infection (*K. pneumoniae*, *Acinetobacter* spp., and *P. aeruginosa*), comorbidities (total number and type), sequential organ failure assessment (SOFA) score, hospital and ICU length of stay (LOS), hospital LOS before ICU admission, and patients' outcome (death or discharge from the ICU). Inflammation and cell damage markers were also recorded: white blood cell count (WBC), C-reactive protein (CRP), D-dimer, and lactate dehydrogenase (LDH). The SOFA score and inflammation and cell damage markers were evaluated after positive cultures of CRGN were confirmed. The criteria for patients' ICU discharge were exactly the same for both groups of patients (COVID-19 and non-COVID-19) by following the hospital's intensive care strategy (not to be needed interventions received in the ICU such as mechanical ventilation, vasoactive medications, renal replacement therapy, and invasive monitoring). In addition, for ICU discharge, patients should not have had clinical and laboratory signs of infection. During their hospitalisation in the ICU, patients with positive CRGN blood cultures who died while presenting clinical and laboratory signs of infection (fever, need for vasoactive medications, increased WBCs, etc.) were considered to die of CRGN infection.

### 2.2. Bacterial Identification and Antimicrobial Susceptibility Testing

Identification of *Gram-negative pathogens was* performed by VITEK® 2 Gram-negative identification cards (bioMérieux, Marcy-l'Etoile, France). Susceptibility to the following antimicrobial agents was studied: amikacin, gentamicin, tobramycin, ciprofloxacin, levofloxacin, imipenem, meropenem, tigecycline, trimethoprim/sulfamethoxazole, and colistin. MICs to ceftazidime, cefepime, ampicillin/sulbactam, piperacillin/tazobactam, and minocycline were evaluated from 2020 to 2023. Antimicrobial agents were selected according to the European Committee for Antimicrobial Susceptibility Testing (EUCAST) guidelines, as well as according to the European Centre for Disease Prevention and Control (ECDC) and the Centers for Disease Control and Prevention (CDC) suggestions [17]. Results were evaluated, and isolates were defined as susceptible (including susceptible and susceptible-increased exposure) and resistant based on EUCAST guidelines [17, 18]. MIC to colistin was determined by the broth microdilution method (SensiTest™ Colistin, Liofilchem, Roseto degli Abruzzi, Italy), as recommended [17].

### 2.3. MDR/XDR/PDR Definitions and Infection Rate

The terms MDR (multidrug-resistant), XDR (extensively drug-resistant), and PDR (pandrug-resistant) were used as described. Specifically, MDR is defined as acquired nonsusceptibility to at least one agent in three or more antimicrobial categories among aminoglycosides, antipseudomonal carbapenems, antipseudomonal fluoroquinolones, antipseudomonal penicillins plus beta-lactamase inhibitors, extended-spectrum cephalosporins, folate pathway inhibitors, penicillin plus beta-lactamase inhibitors, polymyxins, and tetracyclines. XDR was defined as nonsusceptible to at least one agent in all but two or fewer antimicrobial categories (i.e., bacterial isolates remain susceptible to only one or two categories), and PDR was defined as nonsusceptible to all agents in all antimicrobial categories [17]. Isolates that were not eligible for categorization as MDR, XDR, and PDR were assigned as susceptible (S).

In addition, an indicator of the infection rate of the patients during their ICU stay was calculated, with the numerator the number of patients' infections and the denominator the ICU LOS for all patients in both groups.

(1) $$\begin{array}{c} \text{Infection rate}=\frac{\text{NUMBER of GRAM}\left(-\right)\text{ INFECTIONS per PATIENT}\left(\text{Number of Infections}\right)}{\text{ICU LOS}}. \end{array}$$

### 2.4. Statistical Analysis

The normality of the data was evaluated using the Shapiro–Wilk and the Kolmogorov–Smirnov tests with a significance level of 0.05. The categorical variables are presented as frequency matrices and percentages. Continuous variables with normal distribution are presented as the mean and standard deviation (SD), and those without normal distribution are presented as the median and interquartile range (IQR). For the categorical variables, the analysis was performed using Fisher's exact and chi-square tests. Following a normal distribution, continuous variables were assessed with the Student's *t*-test, while those without a normal distribution were tested with the Mann–Whitney *U* test.

Three different analyses were conducted based on a predefined analysis plan. The first analysis aimed to determine the factors that differ among COVID-19 and non-COVID-19 patients. The second analysis aimed to detect predictors of MDR/XDR infections from Gram (−) microorganisms and predictors of patient mortality. In the third analysis, Kaplan–Meier curves and the logrank test were used to compare the overall survival and the survival secondary to CRGN infections of the two groups. To identify predictors of infections from Gram (−) MDR/XDR microorganisms and predictors of patient mortality, a multivariate regression analysis was conducted using logistic regression or cox regression (for mortality) with backward stepwise elimination. Variables with *p*-values<0.1 in the univariate regression were included in the multivariable model, while the choice of variables was based on scientific knowledge and considered potential collinearity. Multicollinearity issues were assessed using the variance inflation factor (VIF).

Independent factors contributing to CRGN infections are presented as the Odds ratio (O.R.) and 95% confidence interval (CI), while independent factors contributing to patients' mortality are presented as the hazard ratio (H.R.) and 95% CI, after checking for the proportionality of hazards. In all cases, *p*-values <0.05 were considered significant. Data were analyzed using the Statistical Package for the Social Sciences (SPSS) (version 28.0; IBM, Armonk, USA) and GraphPad Prism Software (La Jolla, CA, version 10.1.0).

## 3. Results

### 3.1. Differences between COVID-19 and Non-COVID-19 Patients

**Figure 1:**
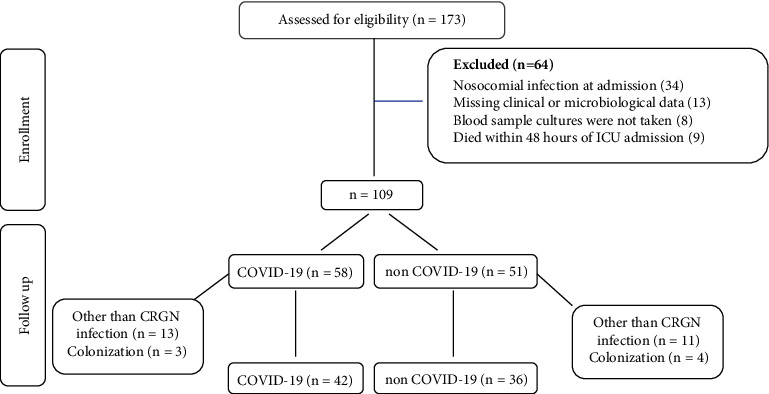
Consort flow diagram.

A total of 173 patients were enrolled, of whom 78 completed the study (42 COVID-19 and 36 non-COVID-19 patients). A modified CONSORT flow diagram, following the STROBE Statement–Checklist, of our study is presented in Figure 1. Differences between the two groups of patients (COVID-19 vs. non-COVID-19) are presented in Table 1. The two groups of patients did not significantly differ in terms of gender and age. Regarding underlying comorbidities, the incidence of obesity was higher in the COVID-19 patients (*p*=0.034), while chronic obstructive pulmonary disease (COPD) was significantly more common in the non-COVID-19 patient group (*p*=0.028). Concerning immunosuppression agents, COVID-19 patients statistically more often received glucocorticosteroids or biological factors compared to non-COVID-19 patients (*p* < 0.001). There were no differences in the incidence of cancer (solid tumors and hematological malignancies) between the two groups (*p*=0.06).

There were no statistically significant differences in the incidence of infections between COVID-19 and non-COVID-19 patients (*p*=0.82). More specifically, 26 (61.9%) COVID-19 and 22 (61.1%) non-COVID-19 patients suffered at least one (range 0–7) episode of infection during their ICU stay (*p*=0.94). Eleven (26.2%) COVID-19 patients and 12 (33.3%) non-COVID-19 patients suffered one episode of infection, 9 (21.4%) COVID-19 patients and 6 (16.7%) non-COVID-19 patients suffered two episodes of infection, 3 (7.1%) COVID-19 and 2 (5.6%) non-COVID-19 patients suffered 3 episodes of infection, and 3 (7.2%) COVID-19 patients and 2 (5.6%) non-COVID-19 patients suffered 4 or more infection episodes. Regarding the specific aetiology of Gram-negative infections, *P. aeruginosa* infections were significantly more common in non-COVID-19 patients (*p*=0.007). Furthermore, there were no statistically significant differences between the two patient groups in the infection rate for all microorganisms (*p*=0.103).

In both COVID-19 and non-COVID-19 patients, infections by MDR microorganisms were more common than infections by XDR microorganisms, but there were no statistically significant differences between the two groups of patients (*p*=0.86 for MDR and *p*=0.81 for XDR microorganisms). More specifically, 23 (54.5%) COVID-19 and 19 (45.2%) non-COVID-19 patients suffered infections by MDR bacteria, while 3 (7.1%) COVID-19 and 3 (8.3%) non-COVID-19 patients suffered infections by XDR bacteria.

Regarding inflammation and cell damage markers, the two groups did not differ statistically significantly in WBCs, CRP, and D-dimers. However, patients with COVID-19 infection had statistically significantly higher LDH values than patients without COVID-19 infection (*p*=0.005).

There were statistically significant differences between the two patient groups in the hospital LOS before admission to the ICU. The COVID-19 patients had statistically significantly more prolonged hospitalisation before ICU admission as compared to non-COVID patients (mean ± SD: 16 ± 16 days vs. 7 ± 18 days, respectively, *p*=0.003). On the other hand, ICU LOS was significantly longer in non-COVID-19 patients (mean ± SD: 25 ± 58 days vs. 10 ± 18 days, respectively, *p*=0.005). Also, regarding outcome, patients with COVID-19 infection had statistically significantly higher mortality than non-COVID-19 patients (*p* < 0.001), while mortality secondary to CRGN infections was also higher in COVID-19 compared to non-COVID-19 patients (*p*=0.033). The two groups also differed statistically significantly in the SOFA score (*p* < 0.001), with COVID-19 patients having higher SOFA scores (mean, ±SD of 9.64 ± 2.39 in COVID-19 patients compared to 6.06 ± 2.19 in non-COVID-19 patients).

### 3.2. Predictors of Developing CRGN Infection

Logistic regression with univariate and multivariate analyses showed that male gender, age, ICU LOS, and hospital LOS before ICU admission were independent risk factors for developing CRGN infections (Table 2). More specifically, men presented 28.7% more infections by CRGN microorganisms than women. Also, the probability of developing CRGN microorganism infection increased by 3.7% (95% CI: 0.3–7%) for 1 year of age, while the probability of developing an infection during ICU hospitalisation increased by 43% (95% CI: 30–98%) with increasing patient age of 10 years.

The probability of developing an infection by CRGN microorganism for each additional day of ICU LOS increased by 1.6% (95% CI: 0.1–3.2%). For every 10 days of increased ICU LOS, there was a 17% (95% CI: 1–37%) increased risk of developing infection.

The probability of developing a CRGN infection for each additional day of hospital LOS before ICU admission increased by 4.6% (95% CI: 0.2–9.1%). For every 10 days of increased hospital LOS before ICU admission, there was a 56% (95% CI: 2–138%) increased risk of developing infection. The infection rate was not an independent risk factor for developing CRGN infections (*p*=0.37, *p*=0.129, and *p*=0.81 for *Acinetobacter* spp., *Pseudomonas aur*., and *Klebsiella pn*., respectively).

### 3.3. Predictors of Patient Mortality

**Figure 2:**
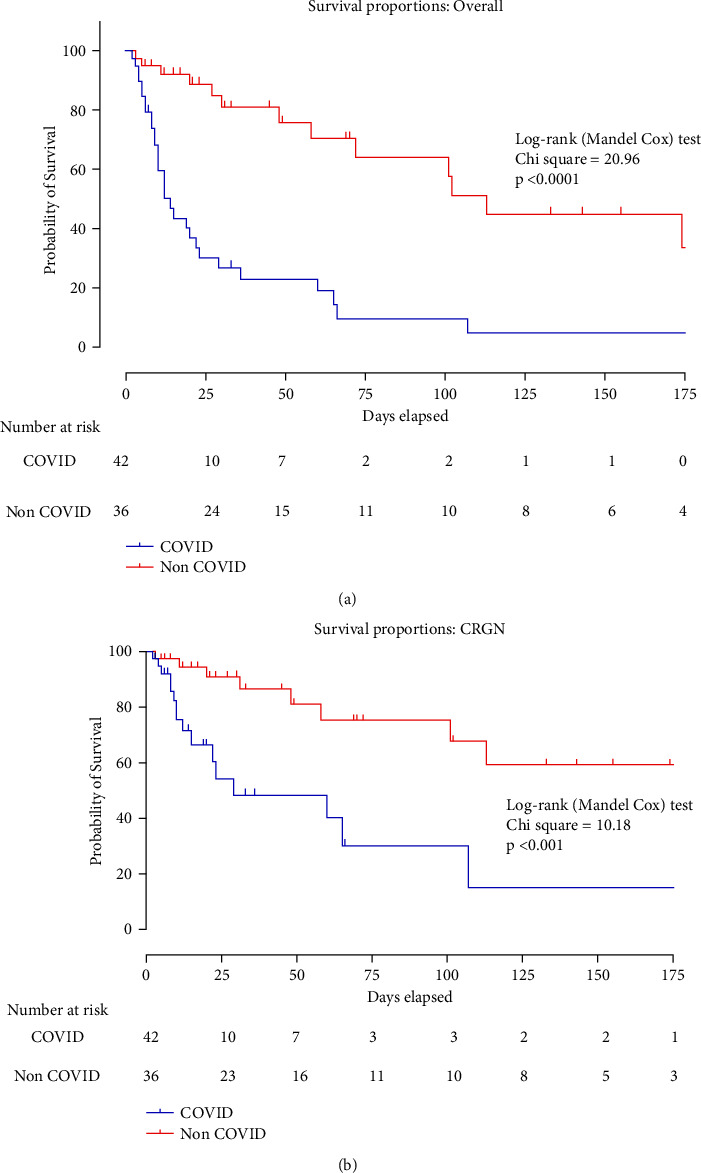
Survival analysis between COVID-19 and non-COVID-19 patients (Kaplan–Meier curves) in all ICU patients who have been analyzed (a) and in patients who died secondary to CRGN infection (b). ICU: intensive care unit; CRGN: carbapenem-resistant gram-negative bacterial infections.

Univariate and multivariate cox regression analysis of data revealed that COVID-19 infection (*p*=0.001), obesity (*p*=0.03), SOFA score (*p*=0.012), the total number of comorbidities (*p*=0.026), absolute WBC count (*p*=0.015), and CRP (*p*=0.015) were independent risk factors for increasing patient mortality. Infection from CRGN pathogens is not an independent risk factor for increased mortality. The results of the univariate and multivariate analyses are presented in Table 3. The Kaplan–Mayer curve and the logrank test (Figure 2) showed that patients with COVID-19 infection had poorer survival rates than patients without COVID-19 infection (logrank test = 20.96 and *p* < 0.0001, Figure 2(a)), while mortality secondary to CRGN infections was also significantly higher in COVID-19 patients compared to non COVID-19 patients (logrank test = 10.18 and *p* < 0.001, Figure 2(b)).

## 4. Discussion

According to the literature, infection with the COVID-19 virus is an independent risk factor for developing immunosuppression and cell damage that increases the host's susceptibility to infections [19, 20]. Regarding the development of bacterial infections in patients with COVID-19 infection, one of the hypotheses is that viral infection causes direct damage to the epithelial cells of the lower airways associated with impaired clearance of mucus, which facilitates the binding of bacteria to cell surfaces [21]. In addition, bacterial superinfection risk in COVID-19 patients is increased due to administration of glucocorticosteroids or biological factors that promote immunosuppression [9]. Previous studies have shown that length of hospital or ICU stay is an important risk factor for acquiring a nosocomial MDRGN infection [22, 23]. Moreover, infections by CRGN pathogens are associated with an increased risk of mortality [24–27]. Therefore, the present study was designed to address the question of contribution of CRGN bacterial infections in the mortality of COVID-19 critically ill patients. Most importantly, to discriminate whether nosocomial-acquired CRGN infections in critically ill COVID-19 patients are associated with the underlying condition of virus- or drug-induced immunosuppression, a comparative group of non-COVID critically ill patients, exposed to the same troubled epidemiological environment of the Greek ICUs, was included.

Our study results showed that the incidence of CRGN infections did not differ significantly in COVID-19 patients compared to non-COVID-19 patients. It should be considered that in the present study, COVID-19 patients remained hospitalized for a longer period before ICU admission, while non-COVID-19 patients had longer ICU LOS. In line with previous studies, our results showed that both hospital (non-ICU) and ICU LOS represent independent risk factors for developing CRGN infections, and therefore, their impact on nosocomial infection acquisition seems to be counterbalanced in the two patient groups [22, 23].

Administration of immunosuppressants was significantly higher in COVID-19 ICU patients. However, despite this fact, the incidence of CRGN infections in COVID-19 patients has not increased. On the contrary, regarding infections from resistant *Pseudomonas aeruginosa*, more cases were observed in non-COVID-19 patients. A recent study showed a decreased risk of colonization and/or infection with MDR in immunocompromised COVID-19 patients compared with immunocompetent subjects [28]. The authors attributed this finding to the importance of contact precautions measures to prevent cross-transmission of MDR bacteria, which are more frequently applied in immunocompromised patients [29]. In addition, previous studies with critically ill mechanically ventilated COVID-19 patients have shown a lower incidence of VAP caused by MDR strains (23.3%) as compared to patients with flu (38.4%) or no viral infection (33.8%) [29, 30]. Except for solid tumors and hematological malignancies, which were similar between the two groups of patients, many other causes of immunosuppression could drive superinfections and mortality in ICU patients, although very recent data have challenged that common assumption [31]. In our analysis, there was only one patient with hemopoietic stem cell transplantation in the COVID-19 group and one patient with solid organ transplant in the non-COVID-19 group.

In a recent review of published studies on the impact of COVID-19 infection in the incidence of ICU-acquired MDR colonization/infection, the authors concluded that MDR bacterial superinfection or colonization is similar or potentially lower in COVID-19 patients vs. controls [16]. Therefore, according to our results, the higher mortality rate observed in critically ill COVID-19 patients was associated with higher disease severity, as assessed with the SOFA score, and the higher presence of comorbidities in this population and not with CRGN bacterial infections.

This study has some important limitations: First, this is a monocentric study with a low number of patients, which might have influenced the potential to reveal differences in the incidence of CRGN bacterial infections. Second, identification of CRGN pathogens was based on conventional microbiological techniques (cultures), whose sensitivity is dropped in patients receiving antibiotic therapy. However, the present study has some advantages as well: it has a prospective design and includes a control group of non-COVID-19 patients to balance the increased risk of CRGN infection acquisition in the troubled epidemiological environment of Greek ICUs.

## 5. Conclusions

The present prospective study has shown that the incidence of infections with CRGN microorganisms did not differ significantly in critically ill ICU COVID-19 patients compared to non-COVID-19 patients. Increased mortality in the COVID-19 patients was not associated with higher CRGN infections but with higher disease severity and more underlying comorbidities. This study was conducted in a European country with a high incidence of infections from CRGN microorganisms. Therefore, its results should not be generalised to the global population of COVID-19 patients. To establish the contribution of critical COVID-19 infection in the incidence of CRGN infections more safely, prospective studies with larger numbers of patients are needed.

## Figures and Tables

**Table 1:** Differences between the two groups of patients (COVID-19 vs non-COVID-19).

	Patients' group		
	COVID-19 (*n* = 42)	Non-COVID-19 (*n* = 36)	*p* value
Age (mean ± SD)	59.35 ± 12.45	57.16 ± 19.51	0.42
Gender (*n*, %)			0.24
Male	31 (73.8%)	31 (86.1%)	
Female	11 (26.1%)	5 (13.9%)	
Comorbidities (*n*, %)			
Diabetes mellitus	7 (16.6%)	5 (13.9%)	0.39
Hypertension	19 (45.2%)	9 (25%)	0.11
Implantable cardioverter defibrillator	3 (7.1%)	5 (13.9%)	0.61
Obesity	13 (31%)	4 (11.1%)	0.034
Heart failure	3 (7.1%)	2 (5.6%)	0.52
Acute kindey injury	3 (7.1%)	6 (16.7%)	0.61
Chronic obstructive pulmonary disease	1 (2.4%)	6 (16.7%)	**0.028**
Cancer	3 (7.1%)	5 (13.9%)	0.06
Solid tumor	2	3	
Hematological malignancies	1	2	
Liver disease	3 (7.1%)	1 (2.8%)	0.27
Systemic lupus erythematosus	3 (7.1%)	3 (8.3%)	0.88
Glucocorticosteroids/biological factors (*n*, %)	41 (97.6)	12 (33)	**<0.001**
SOFA score (mean ± SD)	9.64 ± 2.39	6.06 ± 2.19	**<0.001**
Infection rate	3.03 ± 11.46	1.42 ± 4.64	0.103
Infection incidence (*n*, %)			
One episode	11 (26.2)	12 (33.3)	0.84
Two episodes	9 (21.4)	6 (16.7)	
Three episodes	3 (7.1)	2 (5.6)	
Four or more episodes	3 (7.2)	2 (5.6)	
Microorganism (*n*, %)			
Klebsiella pneumoniae	16 (38.1%)	12 (33.3%)	0.29
Acinetobacter spp	17 (40.5%)	15 (41.7%)	0.91
Pseudomonas aeruginosa	1 (4.2%)	8 (22.2%)	**0.007**
Markers of inflammation and cell damage (mean ± SD)			
WBCs (absolute number/mm^3^)	27.42 ± 17	21 ± 11	0.05
LDH (U/L)	600 ± 687	425 ± 1840	**0.005**
CRP (mg/dl)	18.19 ± 11.4	20 ± 12.55	0.54
D-dimer (*μ*g/l)	4.39 ± 7	5.5 ± 7	0.77
ICU LOS (days) (median ± IQR)	10 ± 18	25 ± 58	**0.005**
Hospital LOS before ICU admission (days) (median ± IQR)	16 ± 16	7 ± 18.75	**0.003**
ICU mortality	32 (76.2%)	14 (38.9%)	**<0.001**
ICU mortality secondary to CRGN infections	19 (45.2%)	8 (22.2%)	**0.033**

SOFA: sequential organ failure assessment score, WBC: white blood cells, LDH: lactate dehydrogenase, CRP: C-reactive protein, ICU: intensive care unit, LOS: length of stay. Statistically significant differences are presented in bold.

**Table 2:** Univariate and multivariate regression analyses of factors associated with developing infection by CRGN microorganisms.

Independent factors contributing to CRGN infections						
Variable	Univariate regression			Multivariate regression		
	O.R.	95% CI	*p* value	O.R.	95% CI	*p* value
Gender (male/female)	0.286	0.093–0.88	0.025	0.287	0.79–1.05	**0.006**
Age	1.028	1.004–1.064	0.032	1.037	1.003–1.071	**0.033**
Group (COVID-19 vs non-COVID-19)-infection rate	1.32	0.42–3.45	0.26			
Diabetes mellitus	4.167	0.847–20.05	0.057	2.4	0.4–13.3	0.3
Hypertension	1.431	0.68–4.73				
Implantable cardioverter defibrillator	1.179	0.26–5.33	0.83			
Obesity	1.36	0.446–4.16	0.58			
Heart failure	1.047	0.16–6.65	0.962			
Acute kidney injury	1.45	0.33–6.28	0.619			
Chronic obstructive pulmonary disease	0.92	0.192–4.425				
Cancer	0.674	0.127–3.576	0.644			
Comorbidities	1.02	0.4–2.6	0.96			
ICU LOS (days)	1.015	1.001–1.028	0.034	1.016	1.001–1.032	**0.04**
Hospital LOS before ICU admission (days)	1.028	0.996–1.061	0.084	1.046	1.002–1.091	**0.004**

ICU: intensive care unit, LOS: length of stay. Statistically significant values are presented in bold.

**Table 3:** Univariate and multivariate cox regression analyses of factors associated with patients' mortality.

Independent factors contributing to patients' mortality						
Variable	Univariate regression			Multivariate regression		
	H.R.	95% CI	*p* value	H.R.	95% CI	*p* value
Gender (male/female)	1.382	0.45–4.22	0.56			
Age	2.126	1.25–3.86	0.008	2.012	0.77–5.21	0.142
Group (COVID-19 vs non-COVID-19)	7.25	1.76–29.8	0.006	0.151	0.05–0.47	**0.001**
Diabetes mellitus	1.18	0.37–3.71	0.77			
Hypertension	1.42	0.47–4.27	0.52			
Implantable cardioverter defibrillator	2.16	0.6–10.2	0.41			
Obesity	4.23	1.106–15.4	0.038	0.004	0.0004–0.6	**0.03**
Heart failure	2.13	0.40–11.13	0.37			
Acute kidney injury	1.83	0.65–5.12	0.25			
Chronic obstructive pulmonary disease	1.75	0.42–7.2	0.43			
Cancer	1.3	0.45–3.22	0.47			
Number of comorbidities	1.656	1.12–2.42	0.016	2.031	1.06–3.4	**0.026**
ICU LOS (days)	1.002	0.99–1.00	0.531			
Hospital LOS (days)	1.022	0.98–1.05	0.121			
CRGN microorganism infection rate						
*Acinetobacter* spp	1.67	0.72–4.71	0.32			
*Pseudomonas aeruginosa*	2.13	0.37–12.15	0.39			
*Klebsiella pneumoniae*	1.23	0.44–3.15	0.5			
SOFA score	1.838	1.343–2.37	<0.001	2.82	1.24–6.3	**0.012**
WBC (absolute number/mm^3^)	1.084	1.033–1.139	<0.001	1.25	1.05–1.531	**0.015**
LDH (U/L)	1.00	1.00–1.003	0.021	1.004	1–1.008	0.06
CRP (mg/dl)	1.084	1.032–1.15	0.001	1.25	1.043–1.521	**0.015**
D-dimer (mg/l)	1.058	0.967–1.148	0.26			

SOFA: sequential organ failure assessment score, ICU: intensive care unit, LOS: length of stay, WBC: white blood cells, LDH: lactate dehydrogenase, CRP: C-reactive protein. Independent factors contributing to patients' mortality are presented in bold.

## Data Availability

The data used to support the findings of this study are available from the corresponding author upon request.

## Additional Points

*Guarantor Statement*. Diamanto Aretha is the guarantor of the content of the manuscript, including data and analysis. All the authors have read and agreed with the content of the article.
